# Efficient Medical Officers and Low Influenza and Pneumonia Rates

**Published:** 1918-12

**Authors:** 


### EFFICIENT MEDICAL OFFICERS AND LOW INFLUENZA AND PNEUMONIA RATES

The question of the control of infectious diseases in an army unit depends more upon the commanding medical officer than any one else, although without the co-operation of his staff and the line officers associated with him he can accomplish little. If the senior medical officer of a unit has vision, and is a man of action who sees to it that every man under him does his duty, contagious diseases will be kept to a minimum among the soldiers for whose health he is responsible. If the commanding medical officer, from division surgeon down to the C. O. of the smallest unit, is the type who "passes the buck" and does not keep track of, and control his personnel to the last private, there will be lax discipline in matters pertaining to hygiene, and a high incidence of preventable diseases. Of course the co-operation of the line officers is a *sine qua non* for results in controlling communicable diseases; but the tactful, efficient, medical officer will be able to impress regimental and company commanders with the necessity for their support.

It has happened too often that one camp will have a high rate from communicable diseases, while another only a few miles away will have a low non-effective rate from the same causes. In the present influenza pandemic in France, however, it cannot be said that commanding medical officers should be held accountable for the high non-effective rates, but even under such unfavorable conditions intelligent effort and "eternal vigilance" will count.

The writer does not know the names of the authors of "The Pneumonia Catechism" and "Directions for Controlling Epidemic Influenza and Pneumonia", or the commanding officers responsible for their dissemination, but it is quite evident that Efficiency dwells at Hospital Center, Remaucourt, and in Base Section No. 2, S. O. S.; and the prediction is hazarded that, when the winter records have been completed, those units will show low non-effective and death rates from influenza, pneumonia, and other respiratory diseases. If every man in the army were required to know and live up to the teachings of the "Pneumonia Catechism" — and there is every reason why each man in the army should know these simple questions and their answers — and if the "Directions for Controlling Epidemic Influenza and Pneumonia" were carried out in every unit of the American troops in France, the influenza and pneumonia problem would be solved.

Efficiency in sanitation means that lives are saved, and that more fighting men are ready for action. Therefore, efficient medical officers back of the lines who prevent diseases perform heroic service, and they should be decorated with the Croix de Guerre and Distinguished Service Cross as surely as soldiers in battle when they bring in a wounded comrade from the firing line. It may be added that physicians, in treating infections like pneumonia and meningitis, are exposed to danger sometimes as great as if they were in the thick of the battle, and it has occurred only too often that in spite of using every precaution, medical officers have died of diseases which they have contracted in line of duty. Is not such sacrifice as heroic as death from enemy shells?

### A Pneumonia Catechism (Headquarters, Hospital Center, Remaucourt).

Commanding Officers of each organization will report by name to this office within two weeks after arrival in camp, that each officer, nurse and employee of his organization can correctly recite the answers to the questions as given below:

Q. What is the greatest danger to which you are exposed in France?

A. Death from such diseases as Meningitis, Pneumonia, Tonsilitis, Influenza, Measles and Diphtheria.

Q. How can I avoid this danger?

A. (1) By preventing comrades from coughing or breathing in my face.

(2) By preventing comrades from spitting in and around buildings.

(3) By preventing comrades from shutting up building so that the air may get close.

Q. What else shall I do to avoid endangering my comrades?

A. (1) I will turn my head when I cough.

(2) I will hold a handkerchief in my hand over my mouth when I cough.

(3) I will keep my handkerchief and my hands clean at all times.

(4) I will fix up a can in which men in my barracks can spit into a disinfecting liquid.

### Directions for Controlling Epidemic Influenza and Pneumonia. (Hdqrs., Base Section No. 2, S. O. S.)

The following directions are given for the guidance of medical officers in the treatment of the disease (epidemic influenza and pneumonia) and in preventing its spread. The situation is serious, and demands the utmost effort on the part of medical officers, and thorough co-operation on the part of line officers.

(*a*) Early recognition of the first cases with their prompt and complete isolation.

(*b*) Every case should be strictly confined to bed until symptoms have been completely abated. Isolation should be maintained throughout convalescence.

(*c*) The bowels should be evacuated at the beginning of an attack, and liquid or light diet be given.

(*d*) In case of any spread of the disease in an organization, the organization should be quarantined; all pass privileges should be withdrawn; no troops should be sent elsewhere from an affected organization; and any new troops which are introduced into it while the disease prevails should be isolated as far as possible from the rest of the organization.

(*e*) It should be remembered that the disease is highly contagious, that it is probably most readily transmitted by the nasal, pharyngeal and bronchial discharges, especially in coughing, sneezing, and spitting, but that it may also probably be transmitted by blankets, clothing, mess kits and other articles used by patients.

(*f*) Public gatherings such as movie shows, concerts, dances, etc., should be prohibited during an epidemic.

(*g*) The utmost effort should be made to prevent overcrowding of the men in quarters during an epidemic. Where feasible, it is well to put men out of doors to sleep in order to prevent overcrowding.

(*h*) Medical officers are directed to report promptly to the Chief Surgeon of this Base any indications of an incipient epidemic among the troops for which they are responsible.

(*i*) Reports from the French Service de Santé inform us that the disease prevails among the civil population in many localities, notably at Bordeaux, Arcachon, Tarbes, and Bayonne. Commanders of organizations in which the disease does not exist are warned against granting leave to any place where the disease is prevalent, on account of the danger of such men introducing the disease into their organization on their return.

